# Impact of trauma teams on high grade liver injury care: a two-decade propensity score approach study in Taiwan

**DOI:** 10.1038/s41598-023-32760-9

**Published:** 2023-04-03

**Authors:** Wen-Ruei Tang, Chun-Hsien Wu, Tsung-Han Yang, Yi-Ting Yen, Kuo-Shu Hung, Chih-Jung Wang, Yan-Shen Shan

**Affiliations:** 1 Department of Surgery, College of Medicine, National Cheng Kung University Hospital, National Cheng Kung University, Tainan, Taiwan; 2 Division of General Surgery, Department of Surgery, College of Medicine, National Cheng Kung University Hospital, National Cheng Kung University, Tainan, Taiwan; 3 Division of Trauma, Department of Surgery, College of Medicine, National Cheng Kung University Hospital, National Cheng Kung University, 138 Sheng-Li Road, Tainan, Taiwan 704; 4 Institute of Clinical Medicine, College of Medicine, National Cheng Kung University, Tainan, Taiwan

**Keywords:** Gastroenterology, Health care, Medical research

## Abstract

High-grade liver laceration is a common injury with bleeding as the main cause of death. Timely resuscitation and hemostasis are keys to the successful management. The impact of in-hospital trauma system on the quality of resuscitation and management in patients with traumatic high-grade liver laceration, however, was rarely reported. We retrospectively reviewed the impact of team-based approach on the quality and outcomes of high-grade traumatic liver laceration in our hospital. Patients with traumatic liver laceration between 2002 and 2020 were enrolled in this retrospective study. Inverse probability of treatment weighting (IPTW)-adjusted analysis using the propensity score were performed. Outcomes before the trauma team establishment (PTTE) and after the trauma team establishment (TTE) were compared. A total of 270 patients with liver trauma were included. After IPTW adjustment, interval between emergency department arrival and managements was shortened in the TTE group with a median of 11 min (*p* < 0.001) and 28 min (*p* < 0.001) in blood test reports and duration to CT scan, respectively. Duration to hemostatic treatments in the TTE group was also shorter by a median of 94 min in patients receiving embolization (*p* = 0.012) and 50 min in those undergoing surgery (*p* = 0.021). The TTE group had longer ICU-free days to day 28 (0.0 vs. 19.0 days, *p* = 0.010). In our study, trauma team approach had a survival benefit for traumatic high-grade liver injury patients with 65% reduction of risk of death within 72 h (Odds ratio (OR) = 0.35, 95% CI = 0.14–0.86) and 55% reduction of risk of in-hospital mortality (OR = 0.45, 95% CI = 0.23–0.87). A team-based approach might contribute to the survival benefit in patients with traumatic high-grade liver laceration by facilitating patient transfer from outside the hospital, through the diagnostic examination, and to the definitive hemostatic procedures.

## Introduction

Trauma is a major healthcare issue worldwide and remains one of the leading causes of death in youth population^1,2^. Specifically, high-grade liver laceration is a common injury with high mortality in blunt trauma^3–5^, with bleeding as the main cause of death. Timely resuscitation, diagnosis and hemostasis are keys to the successful management for patients with high-grade liver injury. A team-based approach considerably improves both the quality and efficiency of resuscitation and increases the survival rate of bleeding trauma patients. The establishment of an in-hospital trauma system facilitates a rapid response and is tailored towards multi-trauma patients with improved patient outcome^6–11^. Therefore, time-related quality indicators were recommended as core factors to evaluate the performance of trauma system^12^.

Our institute is a tertiary referral hospital and a designated level I trauma center serving approximately 3000 trauma patients annually. The Joint Commission of Taiwan has accredited the quality of emergency care in hospitals since 2010, when the trauma team in our institution was established to set-up in-hospital trauma system. In Taiwan, there was no formal training program for trauma surgeon yet. The trauma team consists of surgeons from different field including general surgery, plastic surgery, thoracic surgery, cardiovascular surgery, neurosurgery, and orthopedics, responding to all kinds of devastating trauma even though some of them are irrelevant to the on-call surgeon’s specialty. All team members are required to take trauma call and response for trauma team activation for the critical trauma patients that includes most of the high-grade liver laceration patients. Recent studies revealed that setting-up of trauma team did not guarantee improvement of outcome of trauma patients^13,14^. Thus, we attempt to explore if setting of trauma team of surgeons from different fields improves the care quality of high-grade liver laceration patients. In particular, we retrospectively reviewed the impact of team-based approach yet tailored management on high-grade traumatic liver laceration from process related quality indicator to final outcome in our hospital.

## Methods

### Data collection and patient enrollment

This retrospective study was approved by the Institutional Review Board of National Cheng Kung University Hospital (IRB No. B-ER-111–128) and the informed consent was waived. All methods were performed in accordance with the relevant guidelines and regulations. Patients with traumatic liver laceration between 2002 and 2020, with medical record extracted from NCKUH electronic medical recording system, medical charts, and trauma registry database, were enrolled in this retrospective study. Patients admitted between January 2002 and August 2010 were classified as “pre-trauma team era (PTTE)”, while those admitted between September 2010 and December 2020 were classified as “trauma team era (TTE)”. Patients aged 16 years and below, with liver injury grades I and II (American Association for the Surgery of Trauma liver injury scale, AAST), incomplete study, admission for other indications and association with severe (Abbreviated Injury Scale, AIS = 5) head, thoracic, or other abdominal injuries leading to death within 48 h, were excluded from the study.

### Trauma conference and quality improvement of trauma care

The trauma conference in our hospital was held as multidisciplinary discussion on a weekly basis, participated by surgeons, emergency physicians, and interventional radiologists. Conclusions or consensus from the conference were translated into workflow modification and protocol revision. Any deviation from the standard of care will be uncovered during the conference to prevent it from happening again.

### Outcomes

The primary outcomes were time-related quality indicators and resuscitation quality, defined as red blood cells to fresh frozen plasma ratio (RBC/FFP ratio) between 0.5 to 1.5^15–18^. Time-related quality indicators included time to first blood laboratory report, time to computed tomography (CT) scan from emergency department (ED) admission, and time to operation room or angiography room for hemostasis of liver injury.

Secondary outcomes includes hospital-free days to Day 90, ICU-free days to Day 28, death within 72 h after arrival, and in-hospital mortality. In PTTE, patients with severe liver injury might have early mortality. This would affect the result of length of hospital stay and ICU stay. To correct the bias, we applied hospital-free days and ICU-free days instead. Hospital-free days to Day 90 was calculated as 90 days minus the length of hospital stay. ICU-free days to Day 28 was calculated as 28 days minus the length of ICU stay. For non-survivors, the hospital-free days and ICU-free days were assigned zero^19^.

### Statistical analyses

Since the two populations were neither matched nor randomized, inverse probability of weighting (IPTW) was applied to balance initial characteristics and severity of injury before and after trauma team establishment. Binary logistic regression model was implemented to estimate the probability of a patient visiting ED with or without trauma team approach.

For primary outcomes, a total of 15 presenting variables were included in propensity score (PS) model: sex, age, comorbidity (Charlson comorbidity index, CCI), transferal from other hospitals, trauma mechanism, shock index (SI), Glasgow coma scale (GCS), liver AAST injury grade, and AIS of different body regions. For secondary outcomes, PS model consisted of factors associated with trauma mortality in literature, including sex, age, CCI, trauma mechanism, SI, GCS, AIS of head and chest, liver AAST injury grade, index treatment for liver laceration, tranexamic acid (TXA) use, transfusion quality, and red blood cell (RBC) resuscitation volume. Each observation was weighted with inverse probability of a patient visiting ED before or after trauma team establishment. Diagrams of propensity score distribution were illustrated in supplementary figures (Online Resources 1 and 2, respectively).

Non-normally distributed continuous variables were presented as median [interquartile range, IQR] and were analyzed using Mann–Whitney U test. Categorical variables were calculated using Pearson’s chi-squared test and Fisher exact test. Median differences were evaluated by Hodges-Lehmann estimator. *P* values of < 0.05 was considered to indicate statistical significance. Statistical analyses were performed using SPSS version 20.0 ((IBM Corp., Armonk, NY, USA).

### Ethical approval and informed consent

This study was approved by Institutional Review Board of National Cheng Kung University Hospital (IRB No. B-ER-111-128) and the informed consent was waived.

## Results

**Figure 1:**
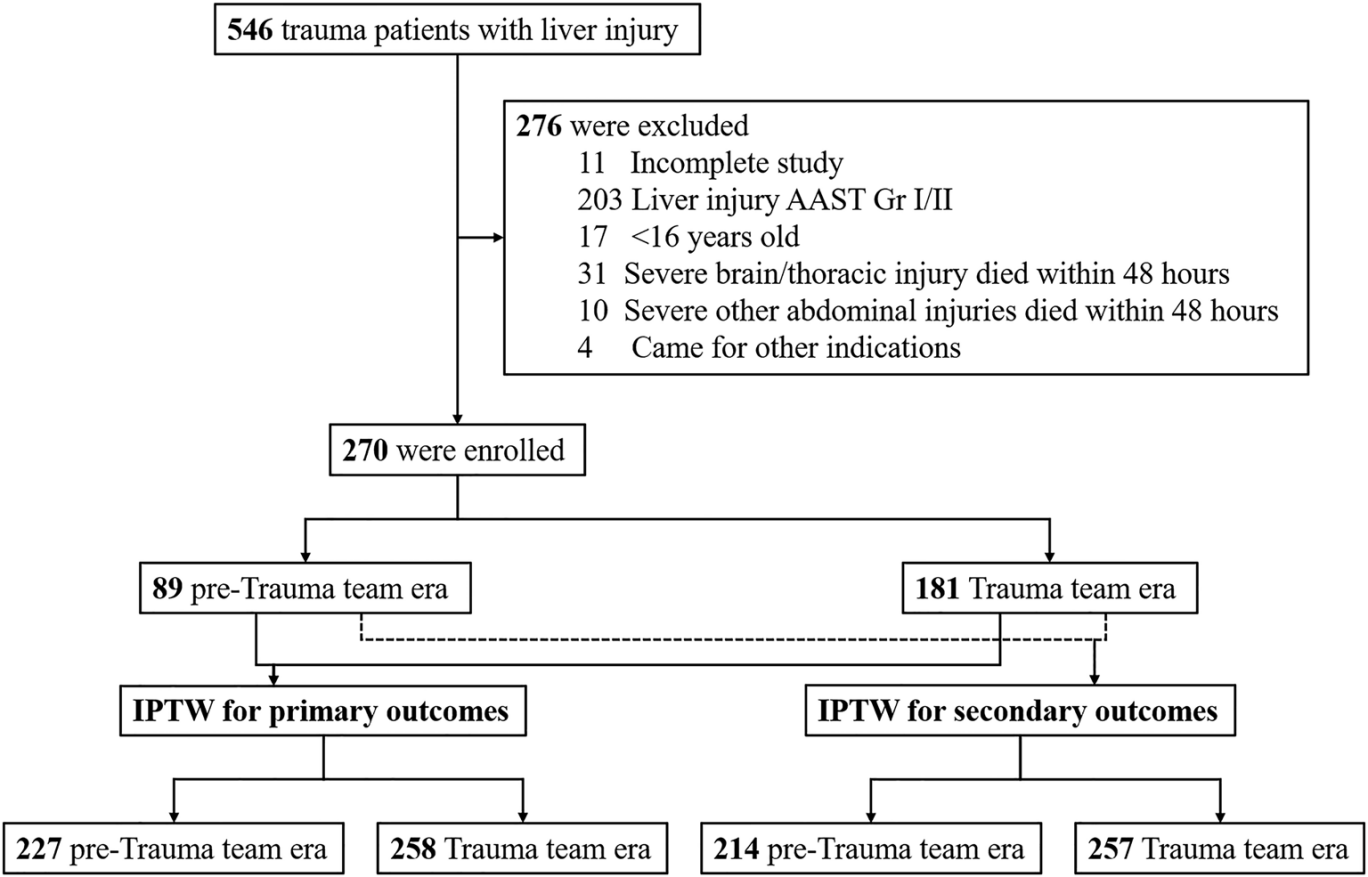
Diagram of study stratification according to propensity score weighting.

A total of 546 patients met the inclusion criteria. We excluded 276 patients and detailed reasons for exclusion were described in Fig. 1. Overall, 270 patients were included, of whom 89 and 181 were classified into the pre-trauma team era (PTTE) group and trauma team era (TTE) group, respectively.

Baseline characteristics of the two groups were shown in Table 1. Prior to inverse probability treatment weighting (IPTW) adjustment, there was no significant difference in age, sex, CCI, trauma mechanism, GCS level, SI $\ge$ 0.9, and cardiac arrest between the two groups. The PTTE group had more patients transferred from other hospitals (51.7% vs. 37.6%, *p* = 0.027), more patients with grade III liver laceration (61.8% vs. 44.8%, *p* = 0.028), and less patients with extremity AIS > 3 (0% vs. 6.1%, *p* = 0.018). There were more patients who received TXA administration (27.0% vs. 46.6%, *p* = 0.002) and non-operative management (NOM) with transcatheter embolization (TAE) (7.9% vs. 50.3%, *p* < 0.001) in TTE group. For the patients needing more than 10 units of RBC transfusion, the TTE group had more patients with optimal RBC/FFP ratio (41.2% vs. 70.2%, *p* = 0.029). After IPTW adjustment for primary outcomes, both groups were comparable except for injury severity of extremity (*p* < 0.002). The weighted characteristics for secondary outcomes were provided in supplementary Table (online Resource 3).

**Table 1:** Initial characteristics.

	Before IPTW^a^		*p*	After IPTW		*p*
	Pre-trauma team N = 89 (32.9)	Trauma team N = 181 (67.1)		Pre-trauma team N = 227 (46.8)	Trauma team N = 258 (53.2)	
Male (%)	41 (46.1)	106 (58.6)	0.053	120 (53.1)	137 (53.1)	0.999
Age, y (median [IQR])	32.0 [23.0–45.5]	33.0 [21.0–49.5]	0.830	29.0 [24.0–49.0]	32.0 [21.0–49.0]	0.868
CCI (median [IQR])						
0	73 (82.0)	129 (71.3)	0.079	183 (80.6)	194 (75.2)	0.060
1 ~ 2	14 (15.7)	37 (20.4)		40 (17.6)	49 (19.0)	
≥ 3	2 (2.2)	15 (8.3)		4 (1.8)	15 (5.8)	
Trauma mechanism						
Blunt (%)	88 (98.9)	176 (97.2)	0.667	224 (99.1)	253 (98.1)	0.457
Penetration (%)	1 (1.1)	5 (2.8)		2 (0.9)	5 (1.9)	
GCS (median [IQR])	15 [14–15]	15 [13–15]	0.258	15.0 [14.0–15.0]	15.0 [14.0–15.0]	0.246
Shock index ≥ 0.9	32 (36.8)	73 (41.2)	0.486	80 (35.4)	92 (35.7)	0.952
Cardiac arrest	6 (6.7)	13 (7.2)	0.894	9 (4.0)	11 (4.3)	0.877
Transferal (%)	46 (51.7)	68 (37.6)	0.027*	94 (41.6)	116 (45.0)	0.456
Liver injury, AAST						
III (%)	55 (61.8)	81 (44.8)	0.028*	131 (58.0)	137 (53.1)	0.524
IV (%)	23 (25.8)	72 (39.8)		72 (31.9)	89 (34.5)	
V (%)	11 (12.4)	28 (15.5)		23 (10.2)	32 (12.4)	
Injury score						
Head AIS > 3	1 (1.1)	9 (5.0)	0.173	11 (4.9)	7 (2.7)	0.212
Face AIS > 3	0 (0.0)	2 (1.1)	> 0.999	0 (0.0)	2 (0.8)	0.501
Thorax AIS > 3	14 (15.7)	36 (19.9)	0.408	31 (13.7)	40 (15.5)	0.579
Extremity AIS > 3	0 (0.0)	11 (6.1)	0.018*	0 (0.0)	11 (4.3)	0.002*
External AIS > 3	0 (0.0)	2 (1.1)	> 0.999	0 (0.0)	1 (0.4)	0.349
Tranexamic acid use (%)	24 (27.0)	84 (46.6)	0.002*	46 (20.3)	131 (50.8)	< 0.001**
RBC/FFP ratio (0.5 ~ 1.5, RBC ≥ 10u)	7 (41.2) N = 17	40 (70.2) N = 57	0.029*	16 (43.2) N = 37	45 (73.8) N = 61	0.003*
Management						
NOM (%)	72 (80.9)	152 (84.0)	0.527	191 (84.5)	230 (89.1)	0.131
NOM without TAE	65 (73)	61 (33.7)		175 (77.4)	97 (37.6)	
NOM with TAE	7 (7.9)	91 (50.3)		16 (7.1)	133 (51.6)	
OM (%)	17 (19.1)	29 (16.0)		35 (15.5)	28 (10.9)	

*AIS* abbreviated injury scale; *CCI* Charlson comorbidity index; *ISS* injury severity score; *NOM* non-operative management; *OM* operative management; *TAE* transarterial embolization.

^a^IPTW model includes sex, age, comorbidity, transferal from other hospitals, trauma mechanism, shock index, Glasgow coma scale, liver AAST injury grade, injury score of different body regions.

* *p* < 0.05.

**Figure 2:**
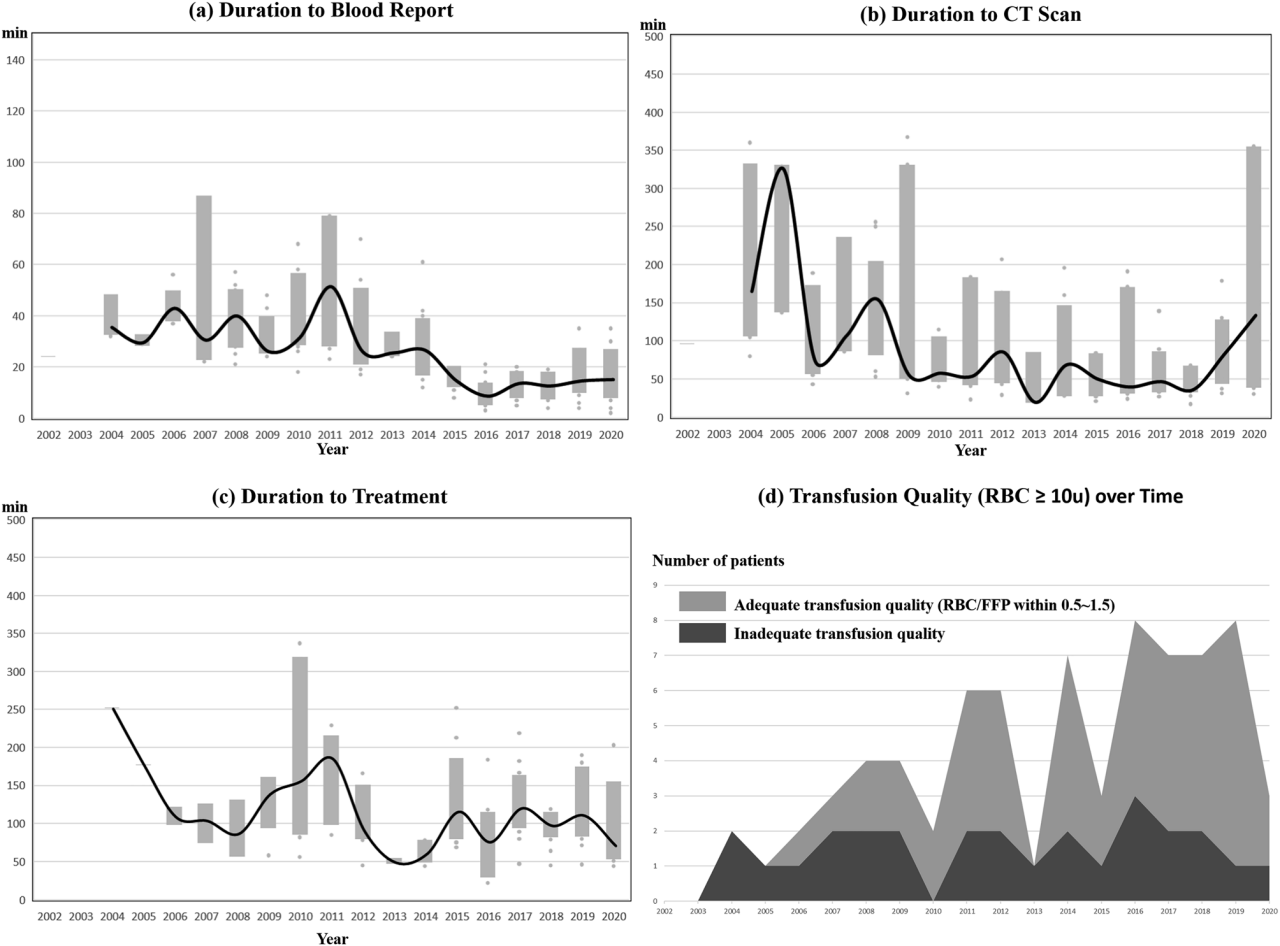
A range of intervals between ED arrival and managements over time. Duration to blood test reports (left upper). Duration to CT scan (right upper). Duration to treatment (left lower). Median curves are depicted. Shaded vertical bars represent interquartile range. Massive transfusion (transfused more than 10 units) quality (percentage of RBC/FFP ratio within 0.5–1.5) over time is shown in right lower panel.

**Figure 3:**
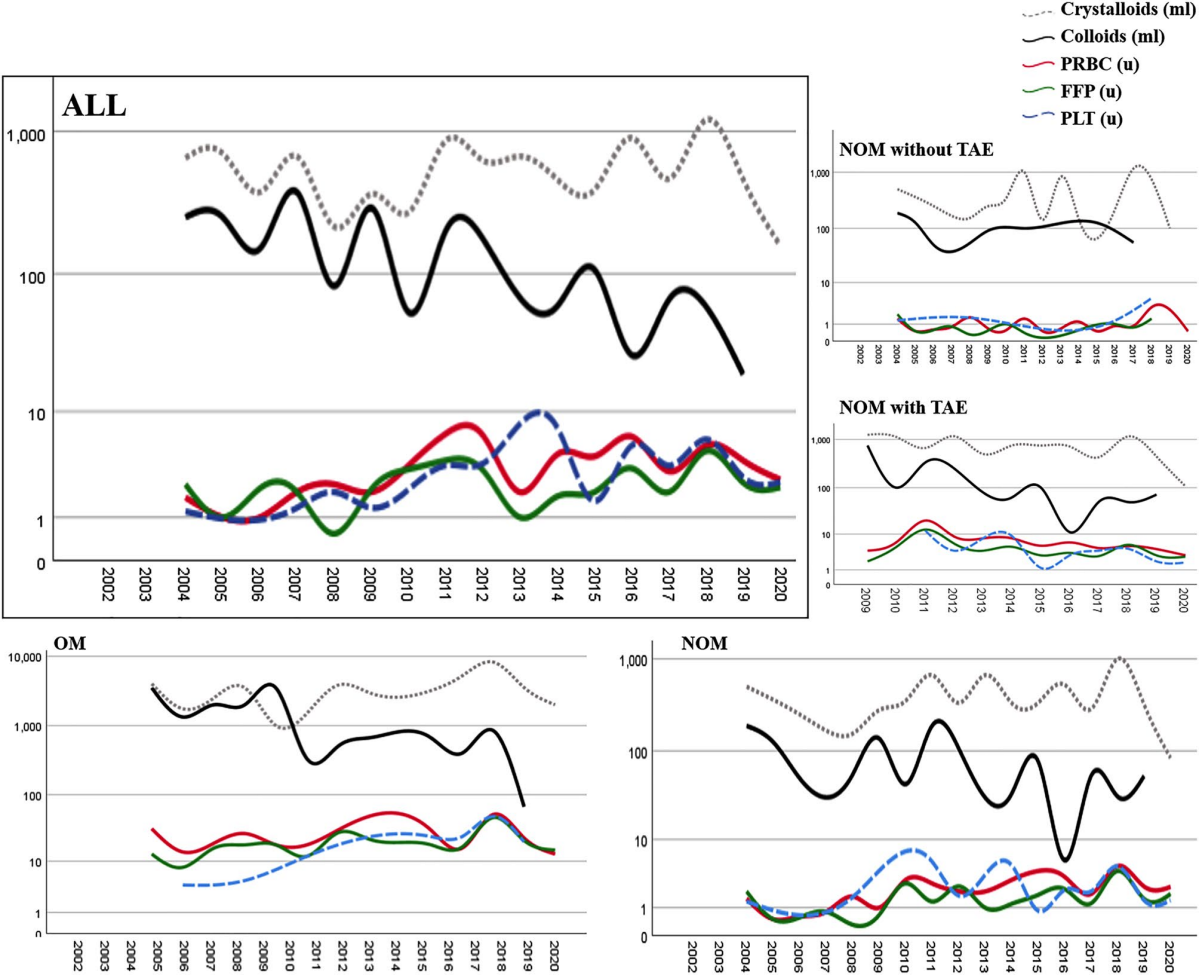
Chronological changes in transfusion amount. Median of resuscitation volume over time. Resuscitation volumes are further stratified by managements. All patients (left upper). NOM without TAE group (right upper). NOM with TAE (right middle). OM (left lower). NOM (right lower).

Table 2 showed the quality indicators of PTTE and TTE groups, before and after IPTW adjustment. In TTE group, time span from patient’s arrival to blood test reports (36 vs. 22 min, *p* < 0.001), duration to CT scan (106 vs. 54 min, *p* < 0.001) and duration to operative management (OM) (135 vs. 67 min, *p* = 0.009) were shorter. After IPTW adjustment, intervals between ED arrival and managements remains markedly shortened, saving a median of 11 min (95% CI = 8–14 min, *p* < 0.001), and 28 min (95% CI = 15–48 min, *p* < 0.001) in blood test reports and duration to CT scan, respectively. Duration to hemostatic treatments was shorter as well by a median of 94 min in patients receiving TAE (95% CI = 16–230 min, *p* = 0.012) and 50 min in those undergoing OM (95% CI = 8–84 min, *p* = 0.021). We excluded transferred patients and compared duration to blood report, CT scan, TAE, and OM between the PTTE and TTE groups. Results presented in Table S2 showed that the TTE group still had significantly shorter durations for these outcomes compared to the PTTE group. Dynamic change of time-related quality indicators was demonstrated chronologically in Fig. 2. The duration to blood test reports, CT scan and treatment gradually decreased after establishment of trauma team. For patients receiving OM, the TTE group had more platelet transfusion and less colloids use. Furthermore, patients in TTE group received more FFP and platelet transfusion and less colloids transfusion after adjustment. Chronological changes in transfusion quality and amount were delineated in Figs. 2d and 3, respectively.

**Table 2:** Primary outcomes.

	Before IPTW^a^		*p*	After IPTW		*p*	Median difference	95% CI	
	Pre-trauma team N = 89 (32.9) (median [IQR])	Trauma team N = 181 (67.1) (median [IQR])		Pre-trauma team N = 227 (46.8) (median [IQR])	Trauma team N = 258 (53.2) (median [IQR])			L	U
Duration to blood report, min	36 [26–49] N = 63	22 [15–35] N = 180	< 0.001*	32 [27–45] N = 146	22 [14–38] N = 257	< 0.001*	− 11	− 14	− 8
Duration to CT scan, min	106 [57–258] N = 65	54 [31–160] N = 122	< 0.001*	105 [48–304] N = 173	63 [31–164] N = 164	< 0.001*	− 28	− 48	− 15
Duration to treatment									
To TAE, min	192 [58–431] N = 7	107 [74–183] N = 91	0.204	235 [121–431] N = 16	107 [74–170] N = 135	0.012*	-94	− 230	− 16
To OM, min	135 [87–177] N = 17	67 [44–108] N = 29	0.009*	135 [42–176] N = 35	80 [44–111] N = 28	0.021*	− 50	− 84	− 8
OM resuscitation	N = 17 (37.0)	N = 29 (63.0)		N = 35 (55.6)	N = 28 (44.4)				
PRBC, u	20.0 [13.5–30.5]	24.0 [15.0–43.0]	0.322	18.0 [18.0–28.5]	22.4 [14.3–40.0]	0.319	3.0	− 4.0	12.0
FFP, u	12.0 [10.0–19.0]	18.0 [12.0–32.0]	0.082	12.0 [12.0–16.0]	18.0[12.0–34.0]	0.013*	6.0	0.0	14.0
PLT, u	0.0 [0.0–0.0]	24.0 [12.0–24.0]	< 0.001*	[0.0–12.0]	24.0 [12.0–24.0]	< 0.001*	12.0	12.0	24.0
Crystalloid, ml	3000 [875–3975]	3650 [2875–5650]	0.066	3000 [2500–4100]	3286 [1661–4748]	0.774	0	− 700	1100
Colloid, ml	2000 [1250–3250]	0 [0–750]	< 0.001*	2000 [1000–2551]	0 [0–500]	< 0.001*	− 1700	− 2000	− 1500

Significant values are in [bold].

*NOM* non-operative management; *OM* operative management; *PLT* platelet; *TAE* transarterial embolization.

^a^IPTW model includes sex, age, comorbidity, transferal from other hospitals, trauma mechanism, shock index, Glasgow coma scale, liver AAST injury grade, injury score of different body regions.

*p < 0.05.

Unweighted and weighted secondary outcomes were described in Table 3. For patients receiving NOM, although there was no difference for ICU-free days between PTTE and TTE groups (24.0 vs. 23.0 days in TTE after adjustment, *p* = 0.065), they had statistically significantly longer hospital-free days to day 90 with a median difference of 2.0 days (95% CI = 0.0–3.0 days, *p* = 0.029). On the other hand, patients receiving OM also had statistically longer hospital-free days to day 90 with a median difference of 20.0 days (95% CI = 0.0–41.0 days, *p* = 0.008) and longer ICU-free days to day 28 (0.0 vs. 19.0 days, *p* = 0.010) after adjusting for IPTW. In our study, trauma team approach had a survival benefit for traumatic high-grade liver injury patients with an odd of 65% lower death within 72 h (Odds ratio (OR) = 0.35, 95% CI = 0.14–0.86) and an odd of 55% lower in-hospital mortality (OR = 0.45, 95% CI = 0.23–0.87).

**Table 3:** Secondary outcomes.

	Before IPTW^a^		*p*	After IPTW		*p*	Median difference	95% CI	
	Pre-trauma team N = 89 (32.9)	Trauma team N = 181 (67.1)		Pre-trauma team N = 214 (45.4)	Trauma team N = 257 (54.6)			L	U
Hospital-free days to day 90, d									
NOM (median [IQR])	77.0 [70.3–80.8] N = 72	77.5 [69.0–82.0] N = 152	0.868	77.0 [70.0–82.0] N = 186	78.0 [71.0–83.0] N = 222	0.029*	2.0	0.0	3.0
OM (median [IQR])	5.0 [0.0–53.0] N = 17	13.0 [0.0–71.5] N = 29	0.523	0.0 [0.0–47.0] N = 28	45.4 [0.0–72.0] N = 35	0.008*	20.0	0.0	41.0
ICU-free days to day 28, d									
NOM (median [IQR])	24.0 [22.8–25.0] N = 62	23.0 [16.0–24.0] N = 110	0.004*	24.0 [22.0–25.0] N = 165	23.0 [18.0–25.0] N = 154	0.065	0.0	− 1.0	0.0
OM (median [IQR])	[0.0–19.0] N = 17	[0.0–21.0] N = 29	0.584	0.0 [0.0–14.7] N = 28	19.0 [0.0–21.0] N = 35	0.010*	7.0	0.0	19.0

	Pre-trauma team	Trauma team	p	Pre-trauma team	Trauma team	*p*	OR	95% CI	
Death within 72 h (%)	8 (9.0)	13 (7.2)	0.602	16 (7.5)	7 (2.7)	0.017*	0.35	0.14	0.86
In-hospital Mortality (%)	10 (11.2)	19 (10.5)	0.854	26 (12.1)	15 (5.8)	0.016*	0.45	0.23	0.87

*NOM* non-operative management; *OM* operative management; *PLT* Platelet; *TAE* transarterial embolization.

^a^IPTW model includes sex, age, comorbidity, trauma mechanism, shock index, Glasgow coma scale, injury score of head and chest, liver AAST injury grade, treatment for liver laceration, tranexamic acid use, transfusion quality, and RBC resuscitation volume.

*p < 0.05.

## Discussion

Process-related quality indicator is used to evaluate the performance of trauma system and identify the opportunities for improvement^12^. However, only few studies evaluated the impact of trauma team on process-related quality indicator and clinical outcomes of patients with high-grade liver laceration. The current study revealed that set-up of trauma team improves the process-related quality indicator in high grade liver laceration population, and is associated with a 65% decreased mortality risk within 72 h (OR = 0.35, 95% CI = 0.14–0.86) and a 55% a reduced chance of in-hospital mortality (OR = 0.45, 95% CI = 0.23–0.87). Our results indicated that a team-based approach might contribute to the survival benefit in patients with traumatic high-grade liver laceration by facilitating patient transfer from outside the hospital, through the diagnostic examination, and to the definitive hemostatic procedures.

A delay in hemostasis for bleeding trauma patients increases the risk of mortality, even in those with stable hemodynamics^20–23^. Early identification of bleeding trauma patients is the first step towards treatment. For patients with liver lacerations, abnormal liver enzymes often indicate the need for a CT scan. In our study, we found that time-to-blood test reports was significantly shorter in the TTE group when weighted for presenting severity and baseline comorbidity. Median time-to-CT scan was reduced by approximately 30 min compared to routine ED care, indicating more effective resuscitation, initial evaluation, and interdisciplinary teamwork. The transferred patients may have different investigation and treatment pathways compared to patients not transferred from other hospitals. To address this, we excluded transferred patients and compared duration to blood report, CT scan, TAE, and OM between the PTTE and TTE groups. The result was similar to the population not excluded transferred patients. Furthermore, Fig. 2 revealed that the time-related quality indicator improved dramatically after the establishment of the trauma team. These findings support our hypothesis that a trauma team can facilitate the initial response to high-grade liver laceration patients.

Management of liver trauma has evolved during the last 30 years with the introduction of modern therapeutic tools and knowledge of damage control surgery^24–26^. In 1995, a prospective trial aiming to evaluate NOM strategies in high-grade liver trauma was released and NOM was proposed as the treatment of choice for hemodynamically stable patients regardless of the severity of the injury^27^. Innovation of endovascular therapy in liver trauma has broadened the fields of NOM. Since the first introduction of TAE in our institute in September 2009, liver trauma management algorithm has been modified. This accounted for the dramatically increased application of TAE in the trauma team era. However, the ratio of operation management was not lowered in TTE when compared with PTTE. This could be due to the fact that TTE group had more patients with grades IV and V liver laceration mandating immediate operation. There were fewer transferred patients in the TTE group, indicating that there were more unstable patients triaged to trauma center after the implementation of trauma team and this group of patients were candidate for laparotomy. Additionally, the patients receiving OM had statistically longer hospital stay in the TTE group after adjustment; nevertheless, they had statistically significantly longer hospital-free days to day 90 with a median difference of 20 days, suggesting that severely injured patients who might have died after damage control surgery (misinterpreted to shorter length of hospital stay) in PTTE would survive to discharge in TTE.

Most trauma deaths occur within 24 h from hemorrhagic shock and the pivotal role of lethal triad is well-known^28–30^. However, massive transfusion is a double-edged sword if not transfused properly^28,31–33^. Compared to a 1:1:2 ratio (FFP:PLT:RBC), transfusion in a 1:1:1 ratio was reported to lower death from exsanguination by 24 h in severely injured patients without increased complication^17^. Although the best transfusion ratio in trauma is still debatable, an optimal range of ratio is thought to be achievable. After setting up of the trauma team, the massive transfusion protocol has been revised and focusing on the ratio of blood component transfused. As shown in Fig. 2d, the percentage of optimal transfusion ratio improved remarkably to 50% and even over 80% after trauma team was established for patients who received 10 or more units of RBC during resuscitation. Concurrently, decreased colloids prescription was observed with early availability of plasma and platelets (Fig. 3), which was also described in previous studies^34^.

In terms of damage control resuscitation, the vital role of TXA cannot be overemphasized. Despite being used widely in elective surgery and obstetric conditions^35,36^, it was not until a randomized controlled trial was published in 2010 that TXA gained popularity in trauma. The CRASH-2 trial demonstrated that early administration (within 3 h of injury) of TXA significantly reduced the risk of death due to bleeding in trauma patients^37^. However, under-utilization and delayed administrated of TXA is a real-life issue for patients receiving massive transfusion^38^, which was corroborated by our study. In our series, TXA use has almost doubled since the establishment of trauma team. However, the application rate remains low. Previously, majority of cases received 500 mg initial dose instead of a 1 g loading dose. However, TXA usage rate increased in the recent five years (Online Resource 4), which was attributed to the revision of massive transfusion protocol in 2018 wherein we set TXA 1 g as standard dose for the bleeding patients.

Trauma team can rapidly respond to critical trauma patients. The team member can arrive at trauma bay within 10 min after trauma team activation. This partially explain why time to operation and time to embolization were significantly decreased in TTE. Delivery of critical trauma patients to operation room and angiography room involves not only the trauma surgeons but also the emergency room nurses, physicians, anesthesiologists, operation room nurses, and intervention radiologists as active members of the care team. Shortening the time to intervention represents a well-functioned team and a well-organized system. In reality, improving most of the process-related quality indicators (e.g., ratio of blood component, using of TXA, time to CT) requires multidisciplinary approach. Setting of trauma team and co-existing of quality index (QI) program was the foundation of providing constant and high-quality care for trauma patients^13,39,40^. This study revealed that several process-related quality indicators of high-grade liver laceration patients improved after setting up of trauma team, and not solely done by the trauma surgeon in the trauma bay. These results support that setting up of a trauma team not only improve rapid response to major trauma patients, but also organize in-hospital system, and provide constant high-quality care for trauma patients.

### Limitations

Our study has a number of limitations that should be acknowledged. Firstly, the study was retrospective and had a limited sample size. Additionally, the long cohort period may have introduced chronological bias, and changes in interdisciplinary connections and patient management principles over time may have impacted the results. Furthermore, there were institutional changes, such as the adoption of electronic medical records, improved quality control in blood banking, and advancements in critical care, which are difficult to analyze and might have contributed to improved care quality and outcome. While we made efforts to minimize bias through propensity score analysis, it may still exist. However, our study focused on measuring efficiency-related quality indicators, particularly the first-line response for major trauma patients. The results were heavily influenced by factors such as teamwork, awareness of critical situations, interdisciplinary communication, and coherence of patient management protocol. It is worth mentioning that our data suggests a significant improvement in time-related quality indicators after the establishment of the trauma team, as shown in Fig. 2. This improvement suggests that the enhancement in primary outcomes is mainly related to the establishment of the trauma team rather than just the passage of time.

## Conclusion

In this study, setting up of trauma team with co-existing QI program effectively facilitates the processes of liver trauma management. A team-based approach might contribute to the survival benefit in patients with traumatic high-grade liver laceration by facilitating patient transfer from outside the hospital, through the diagnostic examination, and to the definitive hemostatic procedures.

## Supplementary Information

Supplementary Information.

## Data Availability

The data that support the findings of this study are available from National Cheng Kung University Hospital, but restrictions apply to the availability of these data, which were used under license for the current study, and so are not publicly available. Data are however available from the corresponding author upon reasonable request.
