# Supplementary material for: Impact of trauma teams on high grade liver injury care: a two-decade propensity score approach study in Taiwan
Source: Sci Rep. 2023 Apr 3;13:5429. doi: 10.1038/s41598-023-32760-9 (PMC10070483; doi:10.1038/s41598-023-32760-9)
Supplement: Supplementary file 1 — Supplementary Information. [file 41598_2023_32760_MOESM1_ESM.docx]

**Table S1.** **Characteristic of secondary outcomes before and after IPTW**

|  | **Before IPTW** | |  | **After IPTW** | |  |
| --- | --- | --- | --- | --- | --- | --- |
|  | **Pre-Trauma Team**  **N = 89 (32.9)** | **Trauma Team**  **N = 181 (67.1)** | **p** | **Pre-Trauma Team**  **N = 214 (45.4)** | **Trauma Team**  **N = 257 (54.6)** | **p** |
| **Male (%)** | 41 (46.1) | 106 (58.6) | 0.053 | 109 (50.9) | 139 (54.1) | 0.495 |
| **Age, y (median [IQR])** | 32.0 [23.0-45.5] | 33.0 [21.0-49.5] | 0.830 | 31.0 [23.0-45.8] | 33.0 [21.0-48.0] | 0.815 |
| **CCI (median [IQR])** |  |  | 0.079 |  |  | 0.599 |
| **0** | 73 (82.0) | 129 (71.3) |  | 164 (76.3) | 194 (75.5) |  |
| **1~2** | 14 (15.7) | 37 (20.4) |  | 42 (19.5) | 47 (18.3) |  |
| **>=3** | 2 (2.2) | 15 (8.3) |  | 9 (4.2) | 16 (6.2) |  |
| **Trauma mechanism** |  |  | 0.667 |  |  | 0.544 |
| **Blunt (%)** | 88 (98.9) | 176 (97.2) |  | 209 (97.2) | 252 (98.1) |  |
| **Penetration (%)** | 1 (1.1) | 5 (2.8) |  | 6 (2.8) | 5 (1.9) |  |
| **GCS (median [IQR])** | 15 [14-15] | 15 [13-15] | 0.258 | 15 [14-15] | 15 [14-15] | 0.819 |
| **Shock index** | 0.83 [0.69-1.02] | 0.80 [0.64-1.04] | 0.558 | 0.81 [0.66-0.96] | 0.80 [0.65-1.06] | 0.761 |
| **Cardiac arrest** | 6 (6.7) | 13 (7.2) | 0.894 | 10 (4.7) | 9 (3.5) | 0.521 |
| **Liver injury, AAST** |  |  | 0.028 |  |  | 0.277 |
| **III (%)** | 55 (61.8) | 81 (44.8) |  | 126 (58.6) | 135 (52.5) |  |
| **IV (%)** | 23 (25.8) | 72 (39.8) |  | 59 (27.4) | 88 (34.2) |  |
| **V (%)** | 11 (12.4) | 28 (15.5) |  | 30 (14.0) | 34 (13.2) |  |
| **ISS (median [IQR])** | 22 [14-26] | 27 [20-36] | <0.001* | 22 [13-25] | 25 [19-34] | <0.001* |
| **Head AIS >3** | 1 (1.1) | 9 (5.0) | 0.173 | 3 (1.4) | 8 (3.1) | 0.221 |
| **Face AIS >3** | 0 (0.0) | 2 (1.1) | >0.999 | 0 (0.0) | 2 (0.8) | 0.503 |
| **Thorax AIS >3** | 14 (15.7) | 36 (19.9) | 0.408 | 31 (14.5) | 43 (16.7) | 0.505 |
| **Extremity AIS >3** | 0 (0.0) | 11 (6.1) | 0.018* | 0 (0.0) | 14 (5.4) | 0.001* |
| **External AIS >3** | 0 (0.0) | 2 (1.1) | >0.999 | 0 (0.0) | 1 (0.4) | >0.999 |
| **Tranexamic acid use (%)** | 24 (27.0) | 84 (46.6) | 0.002* | 70 (32.6) | 105 (40.9) | 0.063 |
| **PRBC/FFP ratio (0.5~1.5)** | 25 (65.8)  N = 38 | 73 (72.3)  N = 101 | 0.455 | 63 (64.3)  N = 98 | 91 (71.7)  N = 127 | 0.238 |
| **PRBC initial resuscitation, u (median [IQR]; mean**$\pm$SD**)** | 2.0 [0.0-6.0]  6.3$\pm$12.1 | 4.0 [0.0-12.0]  9.7$\pm$14.9 | 0.006* | 2.0 [0.0-6.0]  7.0$\pm$14.1 | 4.0 [0.0-10.0]  7.7$\pm$12.6 | 0.099 |
| **Management** |  |  | 0.527 |  |  | 0.865 |
| **NOM (%)** | 72 (80.9) | 152 (84.0) |  | 189 (86.9) | 222 (86.4) |  |
| **OM (%)** | 17 (19.1) | 29 (16.0) |  | 28 (13.1) | 35 (13.6) |  |
| AIS, abbreviated injury scale; CCI, Charlson comorbidity index; ISS, injury severity score; NOM, non-operative management; OM, operative management; TAE, transarterial embolization | | | | | | |

*p <0.05

**Table S2. Primary outcomes of not-transferred subgroup**

|  | **Before IPTW^a^** | |  | **After IPTW** | |  |  |  | |
| --- | --- | --- | --- | --- | --- | --- | --- | --- | --- |
|  | **Pre-Trauma Team**  **N = 43 (34.6)**  **(median [IQR])** | **Trauma Team**  **N = 113 (63.4)**  **(median [IQR])** | **p** | **Pre-Trauma Team**  **N = 132 (48.2)**  **(median [IQR])** | **Trauma Team**  **N = 142 (51.8)**  **(median [IQR])** | **p** | **Median Difference** | **95% CI** | |
|  |  |  |  |  |  |  |  | **L** | **U** |
| **Duration to blood report, min** | 40 [27–58]  N = 27 | 20 [11–39]  N = 112 | <0.001* | 39 [31–48]  N = 72 | 23 [10–52]  N = 141 | <0.001* | -15 | -19 | -9 |
| **Duration to CT scan, min** | 115 [55–251]  N = 43 | 56 [30–154]  N = 109 | <0.001* | 105 [48–254]  N = 132 | 70 [31–163]  N = 138 | 0.006* | -22 | -40 | -6 |
| **Duration to treatment** |  |  |  |  |  |  |  |  |  |
| **to TAE, min** | 337 [337–337]  N = 1 | 119 [81–200]  N = 56 | 0.211 | 337 [337–337]  N = 2 | 138 [82–193]  N = 19 | 0.032* | -197 | -275 | -85 |
| **to OM, min** | 142 [128–177]  N = 7 | 85 [53–112]  N = 20 | 0.008* | 176 [140–177]  N = 15 | 85 [58–116]  N = 70 | <0.001* | -66 | -94 | -47 |

NOM, non-operative management; OM, operative management; TAE, transcatheter arterial embolization

^a^ IPTW model includes sex, age, comorbidity, transferal from other hospitals, trauma mechanism, shock index, Glasgow coma scale, liver AAST injury grade, injury score of different body regions.

* p < 0.05


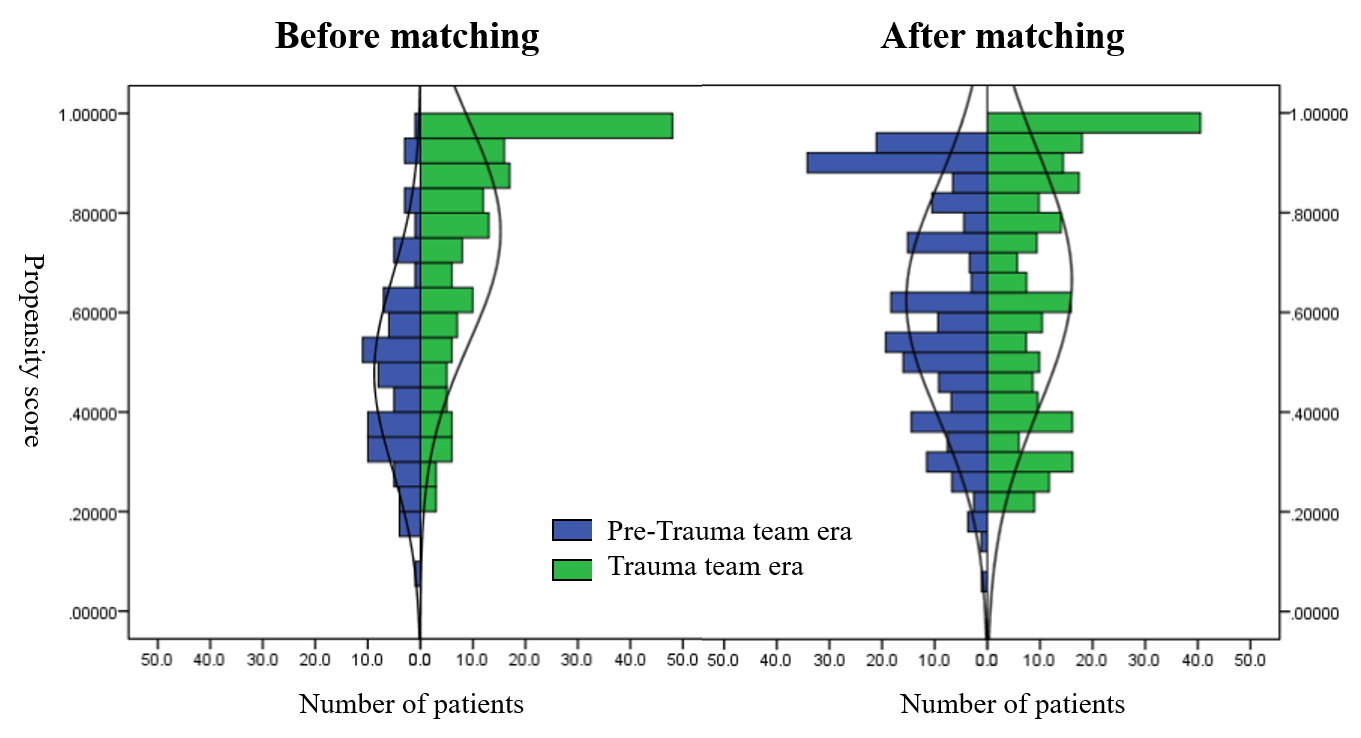


Fig S1. Propensity score density plot for primary outcomes


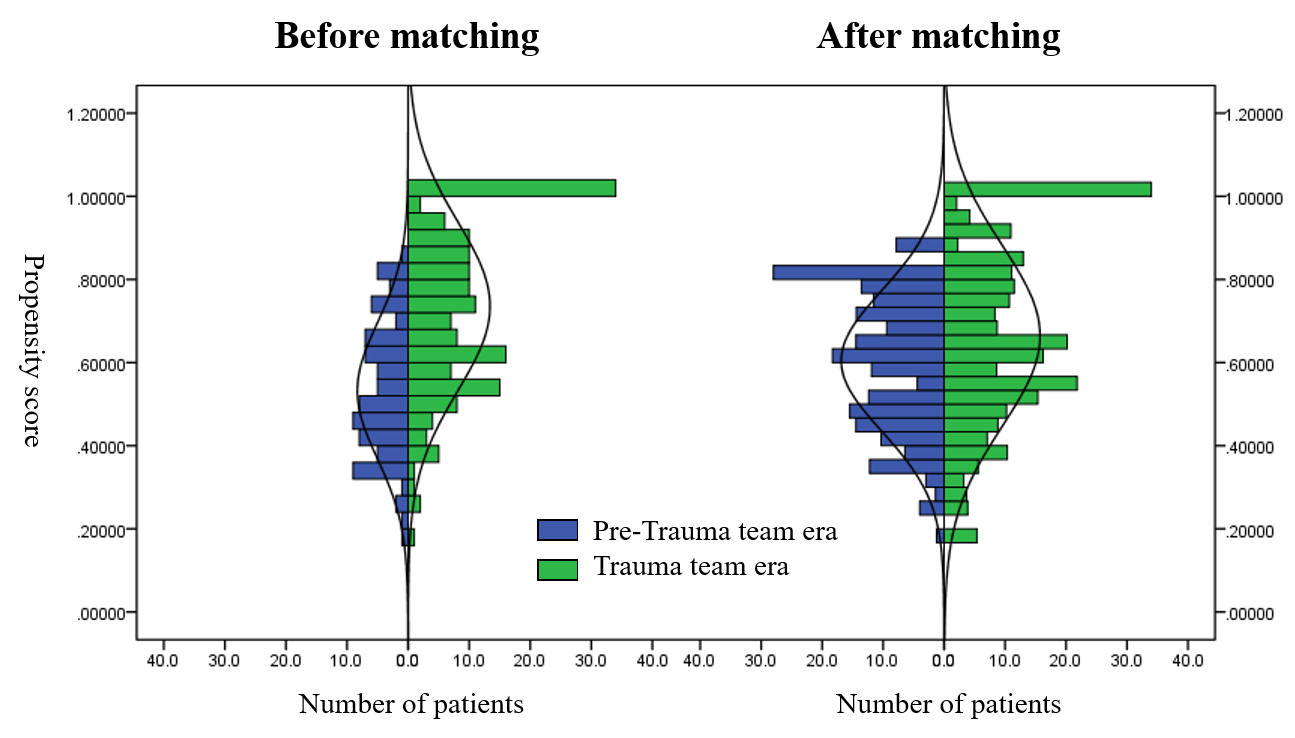


Fig S2. Propensity score density plot for secondary outcomes


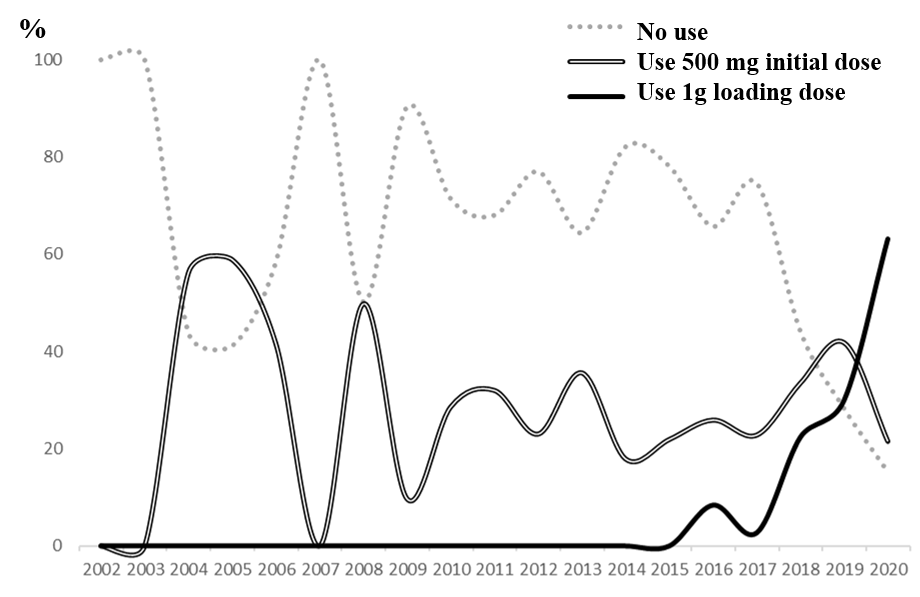


Fig S3. Tranexamic acid use condition over time.
